# Effect of Base–Acid Properties of Mixtures of Ethanol with Water on the Enthalpy of Solution of Cyclic Ethers in these Mixtures at *T* = 298.15 K

**DOI:** 10.1007/s10953-016-0557-8

**Published:** 2016-12-20

**Authors:** Małgorzata Jóźwiak

**Affiliations:** Department of Physical Chemistry, Faculty of Chemistry, University of Lodz, Pomorska 165, 90-236 Lodz, Poland

**Keywords:** Cyclic ethers, Ethanol–water mixtures, Base–acid properties, Enthalpy of solution

## Abstract

The enthalpies of solution of the cyclic ethers 1,4-dioxane, 12-crown-4 and 18-crown-6 in mixtures of ethanol and water have been measured within the whole mole fraction range at *T* = 298.15 K. The enthalpy of solvation has been calculated. In pure ethanol and pure water, the solvation enthalpy of the investigated cyclic ethers depends linearity on the number of –CH_2_CH_2_– groups in the cyclic ether molecules. Based on the analysis of the preferential solvation model proposed by Waghorne, it can be concluded that the 1,4-dioxane, 15C5 and 18C6 molecules are preferentially solvated by water molecules in the range of low water content in these mixtures. The effect of base–acid properties of ethanol–water mixtures on the enthalpy of solution of cyclic ethers in these mixtures has been analyzed. The enthalpy of solution of cyclic ethers correlates with the acidic properties of ethanol–water mixtures in the range of high and medium water content. The results presented are compared with analogous data obtained for the methanol–water and propan-1-ol–water mixtures.

## Introduction

Interactions that occur in solutions are very important for various processes occurring in these solutions. Thinking about the interactions, we refer to the interactions between solute–solute molecules, solvent–solvent molecules and solvent–solute molecules, *i.e.* solvation. The solvation process has been very intensively studied using different methods [1–9].

In the case of a mixed solvent, interactions are observed between the solute molecules and the molecules of the two components of the mixture. In these mixtures, selective solvation of solute molecules may occur. This process is very important and has also been investigated by many scientists [10–17].

A specific group of mixed solvents consists of mixed aqueous–organic solvents because of the very interesting properties of water. In pure water, as well as in mixtures with high water content, the phenomenon of hydrophobic hydration may occur. The properties of solvents have a great impact on the processes of solvation [2–5, 18, 19].

For some time we have been investigating the effect of mixed solvent properties on the enthalpy of solution of cyclic ethers. We have studied the structural–energetic [20, 21] and the acid–base properties of mixed aqueous–organic solvents [22–25].

To characterize the Lewis basicity of the solvent, the Kamlet–Taft parameter *B*_KT_ has been used, while the Lewis acidity has been expressed by the standardized Dimroth–Reichardt parameter $E_{\text{T}}^{\text{N}}$ [26]. In earlier works, mixtures of water (W) and the organic solvents acetonitrile (AN) [22], acetone (ACN) [23], dimethylsulfoxide (DMSO) [23], methanol (MeOH) [24] and propan-1-ol (PrOH) [25] were used for such studies.

The present paper is a continuation of the study on the effect of base–acid properties of the ethanol + water mixed solvent (${\text{EtOH}} + {\text{W}}$) on the solution enthalpy of cyclic ethers in these mixtures. The results obtained are compared with the appropriate data for solutions in MeOH + W and PrOH + W mixtures.

## Experimental

### Materials

Suppliers, purity, method of purification and water contents in the compounds used for measurements (1,4-dioxane, 12-crown-4, 18-crown-6, ethanol) are shown in Table 1.

**Table 1:** Materials

Chemical name	Source	Initial mole fraction purity	Purification method	Mass fraction of water
Urea	Fluka	>0.995^a^	Recrystallization from ethanol and dried under vacuum to constant mass	−
KCl	Sigma–Aldrich	>0.995^a^	Dried under vacuum to constant mass	−
1,4-Dioxane	Aldrich	>0.99^a^	Used as received	2 × 10^−4b^
12C4	Fluka	≥0.98^a^	Dried under vacuum	7 × 10^−4b^
18C6	Fluka	≥0.99^a^	Dried under vacuum	−
EtOH	Chempur	>0.998	Purification and distillation^c^	6 × 10^−4b^

^a^Declared by the supplier

^b^Determined by the Karl Fisher method

^c^Ref. [27]

### Methods

The enthalpy of solution of cyclic ethers in ethanol–water (${\text{EtOH}} + {\text{W}}$) mixtures was measured at (298.15 ± 0.01) K using an “isoperibol” type calorimeter as described in the literature [28]. The calorimeter performance was verified on the basis of the standard enthalpies of solution of urea and of KCl (calorimetric standard US, NBS) in water at (298.15 ± 0.01) K [29, 30] as was described in our recent publication [31]. The value of solution enthalpy in water obtained by us from seven measurements for urea is (15.31 ± 0.06) kJ·mol^−1^ (literature data 15.31 kJ·mol^−1^ [32], 15.28 kJ·mol^−1^ [33] and 15.30 kJ·mol^−1^ [34]) and for KCl is (17.55 ± 0.05) kJ·mol^−1^ (literature data 17.58 kJ·mol^−1^ [29, 30]).

The concentration of cyclic ethers in mixtures was from (0.001 to 0.01) mol·kg^−1^ (mole per kilogram of solvent). Nine to eleven independent measurements were performed for each investigated system. The uncertainties in the measured enthalpies did not exceed ±0.5% of the measured value. No concentration dependence (outside the error limits) of the measured enthalpies of solution was observed within the examined concentration range of cyclic ethers. For this reason, the standard enthalpy of solution $\Delta_{\text{sol}} H^{ \circ }$ was calculated as the mean value of the measured enthalpies (Table 2).

**Table 2:** Standard enthalpy of solution of 1,4-dioxane, 12C4 and 18C6 in the ${\text{EtOH}} + {\text{W}}$ mixtures at *T* = 298.15 K

*x*_w_	$\Delta_{\text{sol}} H^{ \circ }$ (kJ·mol^−1^)		
	1,4-dioxane	12C4	18C6
0.00	6.54 ± 0.06 6.36^a^	3.75 ± 0.04	42.79 ± 0.03 43.11^h^
0.10	5.75 ± 0.03	2.04 ± 0.05	33.81 ± 0.03
0.20	4.92 ± 0.03	0.51 ± 0.06	26.17 ± 0.03
0.30	4.14 ± 0.04	−1.13 ± 0.06	20.85 ± 0.02
0.40	3.54 ± 0.03	−2.60 ± 0.05	17.51 ± 0.04
0.50	3.23 ± 0.05	−3.63 ± 0.04	14.72 ± 0.03
0.60	3.03 ± 0.05	−4.73 ± 0.05	12.23 ± 0.03
0.70	2.89 ± 0.04	−5.81 ± 0.04	10.75 ± 0.04
0.80	2.76 ± 0.03	−7.83 ± 0.05	7.89 ± 0.03
0.90	0.56 ± 0.03	−12.67 ± 0.04	0.57 ± 0.05
0.92	−0.76 ± 0.05	−15.21 ± 0.04	−2.94 ± 0.04
0.94	−2.77 ± 0.03	−18.55 ± 0.03	−7.53 ± 0.03
0.96	−4.96 ± 0.05	−21.89 ± 0.05	−11.99 ± 0.04
0.98	−7.28 ± 0.04	−25.32 ± 0.04	−16.58 ± 0.06
1.00	–9.69 ± 0.03	–28.98 ± 0.06	–21.58 ± 0.04
	–9.70 ± 0.04^b^	–28.98 ± 0.08^d^	–21.58 ± 0.06^i^
	–9.70 ± 0.02^c^	–28.95 ± 0.05^g^	–21.54 ± 0.05^g^
	–9.64 ± 0.05^d^		
	–9.60 ± 0.03^e^		
	–9.34^f^		

^a^Ref. [35]

^b^Ref. [36]

^c^Ref. [24]

^d^Ref. [37]

^e^Ref. [38]

^f^Ref. [39]

^g^Ref. [40]

^h^Ref. [41]

^i^Ref. [42]

## Results and Discussion

Using the standard solution enthalpy data of cyclic ethers, literature data for 15C5 [31], and data of the enthalpy of vaporization of cyclic ethers [43, 44], the standard solvation enthalpies of cyclic ethers in pure ethanol and in pure water were calculated. It is observed that the standard solvation enthalpy of cyclic ethers in EtOH and W depends linearly on the number of –CH_2_CH_2_O– groups ($n_{{ - {\text{CH}}_{ 2} {\text{CH}}_{ 2} {\text{O}} - }}$) in the cyclic ether molecules (Eqs. 1 and 2):

1 $$ \Delta_{\text{solv}} H^{ \circ } = - 14.57 (0.273 )\cdot n_{{ - {\text{CH}}_{ 2} {\text{CH}}_{ 2} {\text{O}} - }} ,{{ R}}^{ 2} = \, 0. 9 9 8 9 6,\;SD = { 2}. 4 40 6,\;P = { 1}. 4 2\times 10^{ - 5} \;{\text{for EtOH}} $$

2 $$ \Delta_{\text{solv}} H^{ \circ } = - 24.25 (0.39 )\cdot n_{{ - {\text{CH}}_{ 2} {\text{CH}}_{ 2} {\text{O}} - }} ,{{ R}}^{ 2} = \, 0. 9 9 9 2 3,\;SD = { 3}. 50 1 7,\;P = { 9}. 1\times 10^{ - 6} \;{\text{for W}} $$

where: *R* is the regression coefficient, *SD* is the standard deviation and *P* is the probability that *R* is equal to 0.

Comparing the coefficients of Eqs. 1 and 2, it can be seen that the contribution of the –CH_2_CH_2_O– group to the standard solvation enthalpy is much higher in water than that in EtOH. This indicates that the interactions of cyclic ether molecules with water molecules are significantly different from the interaction of cyclic ether molecules with molecules of EtOH. This was expected because of the hydrophobic hydration of cyclic ether molecules in water.

For the description of solutions in binary solvent, the enthalpy of transfer ∆_tr_ *H*°, of the solute from an individual solvent (S) to the mixed solvent (${\text{M}} = {\text{S}} + {\text{Y}}$) of various compositions, is a very convenient function. It allows us to very easily compare the effect of the mixed solvent composition change on the behavior of different solutes in the same mixture as well as to compare the effect of the added cosolvent on the properties of solutions of the same solute in different mixed solvent. Moreover, the changes in the transfer enthalpy as a function of solvent composition are the same as those in the enthalpy of solvation.

The transfer enthalpy can be calculated as the difference between the standard enthalpy of solution of the solute in the mixture (M) and in the single solvent (S):

3 $$ \Delta_{\text{tr}} H^{ \circ } = \Delta_{\text{solv}} H^{ \circ } ( {\text{M)}} - \Delta_{\text{solv}} H^{ \circ } ( {\text{S)}} = \Delta_{\text{sol}} H^{ \circ } ( {\text{M)}} - \Delta_{\text{sol}} H^{ \circ } ( {\text{S)}} $$

where: $\Delta_{\text{solv}} H^{ \circ }$ is the standard solvation enthalpy and $\Delta_{\text{sol}} H^{ \circ }$ is the standard enthalpy of solution.

The transfer enthalpy of cyclic ethers from water W to ${\text{EtOH}} + {\text{W}}$ mixtures can be described by Eq. 4:

4 $$ \Delta_{\text{tr}} H^{ \circ } ( {\text{EtOH}} + {\text{W)}} = \Delta_{\text{sol}} H^{ \circ } ( {\text{EtOH}} + {\text{W)}} - \Delta_{\text{sol}} H^{ \circ } ( {\text{W)}} $$

where $\Delta_{\text{tr}} H^{ \circ } ( {\text{EtOH}} + {\text{W)}}$ is the transfer enthalpy of cyclic ethers from water to the ${\text{EtOH}} + {\text{W}}$ mixtures, $\Delta_{\text{sol}} H^{ \circ } ( {\text{EtOH}} + {\text{W)}}$ is the standard enthalpy of solution of the cyclic ethers in the ${\text{EtOH}} + {\text{W}}$ mixtures, and $\Delta_{\text{sol}} H^{ \circ } ( {\text{W)}}$ is the standard enthalpy of solution of cyclic ethers in water.

**Fig. 1:**
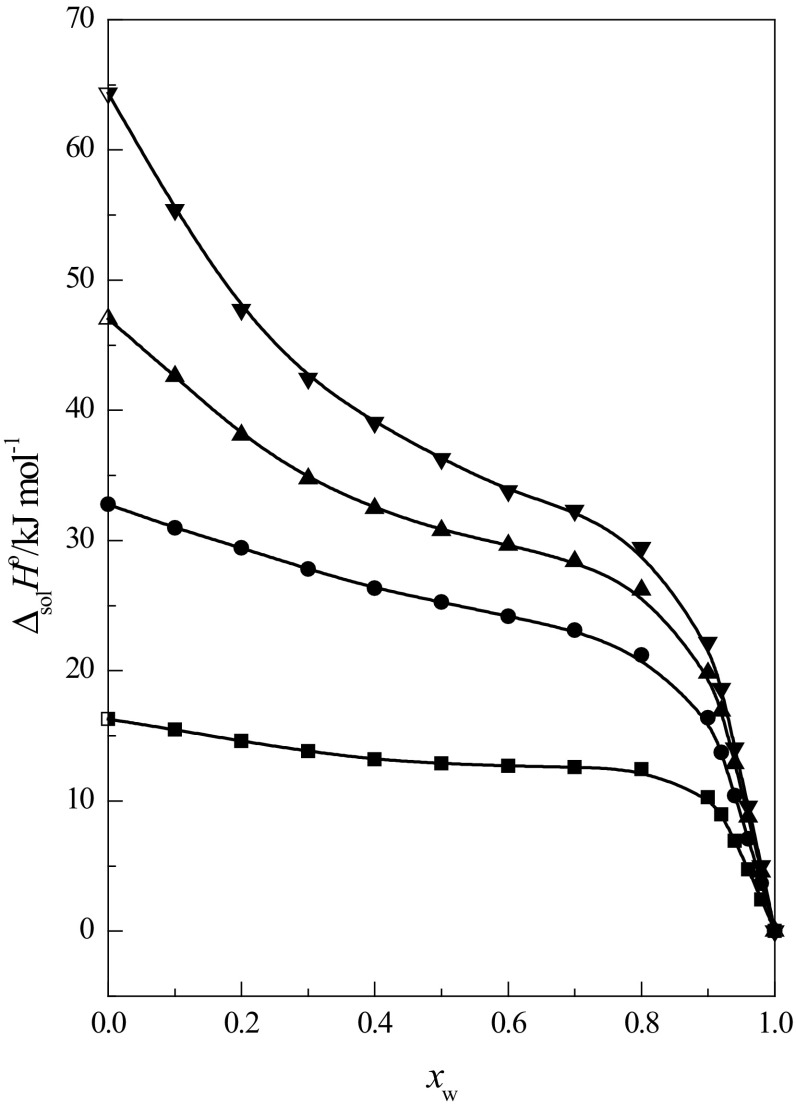
Transfer enthalpy of cyclic ethers: 1,4-dioxane (*filled square*), 12C4 (*filled circle*), and 15C5 (*filled triangle*) (calculated using the standard enthalpy of solution of 15C5 [31]) and of 18C6 (*filled inverted triangle*) from water to ${\text{EtOH}} + {\text{W}}$ mixtures as a function of the water mole fraction (*x*_w_) at 298.15 K

In Fig. 1, the transfer enthalpies of cyclic ethers investigated in this paper are presented as a function of mole fraction of water *x*_w_ in the ${\text{EtOH}} + {\text{W}}$ mixtures. In order to compare the data obtained, the transfer enthalpy of 15-crown-5 ether (15C5) (calculated using the data of the standard enthalpy of solution of 15C5 [31]) was included. As is seen in this figure, the shapes of the transfer enthalpy curves of the cyclic ethers investigated are similar to each other. The values of this function decrease with increasing the concentration of water in the mixtures. With increasing cyclic ring size, the variability of the transfer enthalpy curves as a function of *x*_w_ becomes more expressive. This is connected with the process of hydrophobic hydration of cyclic ethers in the high water region in the mixtures [37]. The hydrophobic hydration can be described as the formation of cages of water molecules around the non-polar molecule, or its non-polar part [45]. This strengthens the structure of water around the solute through hydrogen bonding reinforcement. This causes a sharp increase in the exothermic dissolution process in water–organic solvent mixtures in the high water content region of the mixtures [46–48].

In the high and medium EtOH content region in the mixtures, the change in the $\Delta_{\text{tr}} H^{\text{o}} = f (x_{\text{w}} )$ function is connected (in my opinion) with the structure of the mixed solvents and interactions of cyclic ether molecules (in particular 15C5 and 18C6) with molecules that are components of the mixtures, *i.e.* preferential solvation [31].

In order to test this hypothesis, the preferential solvation model proposed by Waghorne et al. [49, 50] was used. In this model, the transfer enthalpy of the solute, $\Delta_{\text{tr}} H^{ \circ } \left( {{\text{W}} \to {\text{W}} + {\text{Y}}} \right)$, from water (W) to the mixed solvent (W + Y), is described with Eq. 5:

5 $$ \begin{aligned} \Delta_{\text{tr}} H^{ \circ } \left( {{\text{W}} \to {\text{W}} + {\text{Y}}} \right) &= \frac{{px_{\text{y}} }}{{x_{\text{w}} + px_{\text{y}} }}\Delta \Delta H_{ 1 2}^{ \circ } - \frac{\alpha n + \beta N}{{x_{\text{w}} + px_{\text{y}} }}\left( {x_{\text{w}} L_{\text{w}} + px_{\text{y}} L_{\text{y}} } \right) \hfill \\&\quad + \frac{{px_{\text{y}} }}{{x_{\text{w}} + px_{\text{y}} }}\left( {\alpha n + \beta } \right)\left( {\Delta H_{\text{w}}^{ *} - \Delta H_{\text{y}}^{ *} } \right) \hfill \\ \end{aligned} $$

where *x*_w_ and *x*_y_ are molar fractions of W and Y, respectively, $p = \frac{{n_{\text{y}} x_{\text{w}} }}{{n_{\text{w}} x_{\text{y}} }}$, *n*_w_ and *n*_y_ are the number of molecules of W and Y in the first solvation sphere of the solute, *p* is a direct measure of the preferential solvation (*p* < 1 denotes that the solute is preferentially solvated by W, *p* > 1 that the preference is for Y), *αn* is connected with the formation of a cavity in the solvent by the solute, *βN* reflects the change in solvent bonds between the first and further solvation spheres. *L*_w_ and *L*_y_ are relative partial molar enthalpies of W and Y in the mixed solvent, respectively, $\Delta H_{\text{w}}^{ *}$ and $\Delta H_{\text{y}}^{ *}$ are the enthalpies of condensation of W and Y, respectively, while $\Delta \Delta H_{ 1 2}^{ \circ } = (\Delta H_{12}^{ \circ } )_{Y} - (\Delta H_{12}^{ \circ } )_{\text{W}}$ represents the differences between the enthalpies of solute–solvent interactions in pure W and Y.

Waghorne’s model was used to separately analyze the obtained calorimetric data within the range of low, medium and high water contents in the mixtures. The values of *p*, $\alpha n + \beta N$ and $\Delta \Delta H_{ 1 2}^{\text{o}}$ were calculated by the method reported by Waghorne et al. [51]. I have chosen two ranges of compositions of the mixed solvent, *i.e*. the area with low and medium water content (*x*_w_ < 0.9), and the area with a high water content (*x*_w_ ≥ 0.92). The results obtained are listed in Table 3. As is seen, the values of the parameter *p* < 1, which indicates preferential solvation of 1,4-dioxane, 12C4, 15C5 and 18C6 by water molecules over the whole range of the mixture composition. The values of the *p* parameter are lower in the range of high water content then those in the mixtures with low and medium water content. In the case of 12C4, 15C5 and 18C6, the negative values of the $\alpha n + \beta N$ parameter and very high value of the $\Delta \Delta H_{ 1 2}^{ \circ }$ parameter indicates the existence of a hydrophobic hydration process [31], which is also a kind of preferential solvation process. Moreover, a linear dependence of the values the $\alpha n + \beta N$ parameter on the number of –CH_2_CH_2_– groups in 1,4-dioxane, 12C4, 15C5 and 18C6 molecules is observed (Eqs. 6 and 7):

**Table 3:** Values of parameters of Eq. 5 for cyclic ethers in the EtOH + W mixtures

*x*_w_	*p*	$\alpha {\text{n}} + \beta {\text{N}}$	$\Delta \Delta H_{12}^{ \circ }$ (kJ·mol^−1^)	R^2^
1,4-Dioxane				
*x*_w_ < 0.9	0.93	12.34	36.16	0.99956
*x*_w_ ≥ 0.92	0.54	16.40	54.97	0.99994
12C4				
*x*_w_ < 0.9	0.66	20.88	69.71	0.99768
*x*_w_ ≥ 0.92	0.10	–12.67	2042.49	0.99968
15C5^a^				
*x*_w_ < 0.9	0.75	24.79	85.21	0.99980
*x*_w_ ≥ 0.92	0.15	–20.06	1816.09	0.99914
18C6				
*x*_w_ < 0.9	0.71	26.69	100.64	0.99894
*x*_w_ ≥ 0.92	0.24	–32.62	1441.26	0.99998

^a^Data for 15C5 taken from Ref. [31]

for *x*_w_ ≥ 0.92,

6 $$ \alpha n + \beta N = 39.18 ( 4. 3 2 )- 12.10 (0. 9 6 )n_{{ - {\text{CH}}_{ 2} {\text{CH}}_{ 2} - }} ,{{R}}^{ 2} = \, 0. 9 8 7 5 5,SD = { 2}. 8 4 1 3,P = \, 0.00 6 2 $$

and for *x*_w_ < 0.9

7 $$ \alpha n + \beta N = 5.48 (1. 6 3 )+ 3.69 (0.36 )n_{{ - {\text{CH}}_{ 2} {\text{CH}}_{ 2} - }} ,R^{ 2} = \, 0. 9 8 1 2 2,SD = { 1}.0 6 8 6,P = \, 0.00 9 4 $$

On the other hand, the ethanol molecules have some hydrophobic properties because of the presence of the CH_3_CH_2_– group in their structure [47]. This kind of property plays an essential role in the range of high water content in the mixtures.

**Fig. 2:**
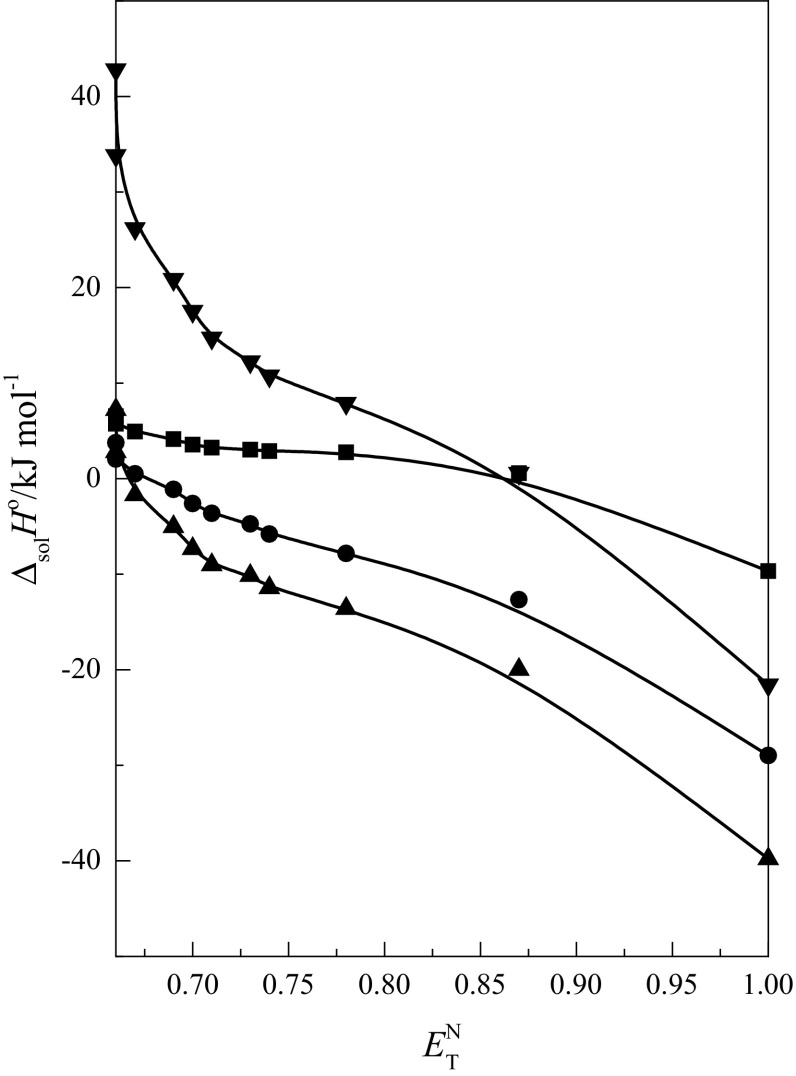
The standard enthalpy of solution of 1,4-dioxane (*filled square*), 12C4 (*filled circle*), 15C5 (*filled triangle*) [31] and 18C6 (*filled inverted triangle*) as a function of acid properties of the ${\text{EtOH}} + {\text{W}}$ mixtures

Analysis of the effect of acid–base properties of the ${\text{EtOH}} + {\text{W}}$ mixtures on the enthalpy of solution of cyclic ethers was also performed. The molecules of cyclic ethers contain oxygen atoms with free electron pairs. This fact causes the cyclic ethers to be regarded as centers of Lewis basicity. For this reason, an analysis was performed with Lewis’ acidity expressed by the standardized Dimroth–Reichardt’s parameter $E_{\text{T}}^{\text{N}}$ for the ${\text{EtOH}} + {\text{W}}$ mixtures [26]. Therefore it was decided to present the enthalpy of solution as a function of $E_{\text{T}}^{\text{N}}$:

8 $$ \Delta_{\text{sol}} H^{ \circ } = Q_{0} + a \cdot E_{\text{T}}^{\text{N}} $$

where $Q_{0}$ is the value of the given property in the absence of the solvent effect, while *a* is the contribution of acidic properties to the variation of the enthalpy of solution. The parameters of the obtained relationship are given in Table 4 (columns labelled a). The functions $\Delta_{\text{sol}} H^{ \circ } ( {\text{EtOH}} + {\text{W)}}$ = *f*($E_{\text{T}}^{\text{N}}$) for 1,4-dioxane, 12C4, 15C5 [31] and 18C6 are shown in Fig. 2.

**Table 4:** Values of parameters of Eq. 8 for cyclic ethers in the ${\text{EtOH}} + {\text{W}}$ mixtures at *T* = 298.15 K

Parameter	1,4-dioxane		12C4		15C5		18C6	
	*a* ^a^	*b* ^b^	*a* ^a^	*b* ^b^	*a* ^a^	*b* ^b^	*a* ^a^	*b* ^b^
*Q*_0_ (kJ·mol^−1^)	32.22 (3.25)^c^	32.47 (4.71)	58.37 (3.64)	56.26 (4.82)	76.68 (7.92)	65.30 (5.92)	128.91 (15.07)	104.03 (6.53)
*a* (kJ·mol^−1^)	−39.79 (4.31)	−40.08 (6.02)	–85.65 (4.84)	–83.20 (6.14)	–115.89 (10.52)	–102.68 (7.55)	–152.53 (20.01)	–123.68 (8.33)
*R* ^2d^	0.90432	0.88087	0.97211	0.96831	0.93094	0.96855	0.86587	0.97352
*SD* ^e^	1.4209	1.7014	1.5923	1.7374	3.4651	2.1357	6.5902	2.3548
*P* ^f^	<0.0001	5.54 × 10^−4^	<0.0001	<0.0001	<0.0001	<0.0001	<0.0001	<0.0001

^a^Parameters of Eq. 8 calculated using the data of standard enthalpy of solution of cyclic ethers for *x*_w_ = 0, 0.1, 0.2, 0.3, 0.4, 0.5, 0.6, 0.7, 0.8, 0.9 and 1

^b^Parameters of Eq. 8 calculated using the standard enthalpy of solution data of cyclic ethers for *x*_w_ = 0.3, 0.4, 0.5, 0.6, 0.7, 0.8, 0.9 and 1

^c^Standard errors are given in the parentheses

^d^ *R* is a regression coefficient

^e^ *SD* is the standard deviation

^f^ *P* is the probability that *R* is equal to 0

It was observed that the *a* parameter linearly increases with the increase in the number of oxygen atoms ($n_{{ - {\text{O}} - }}$), which is the same as the number of –CH_2_CH_2_O– groups ($n_{{ - {\text{CH}}_{ 2} {\text{CH}}_{ 2} {\text{O}} - }}$) in the cyclic ether molecules (Eq. 9). Standard deviations are given in parentheses.

9 $$ a = { 19}. 5 2\left( { 10. 2 4} \right){-} 2 7. 7 6\left( { 2. 2 8} \right) \cdot n_{{ - {\text{O}} - }} ,{{ R}}^{ 2} = \, 0. 9 8 6 7 5,SD = { 6}. 7 2 9 2,P = { 6}. 6 5\times 10^{ - 3} $$

**Fig. 3:**
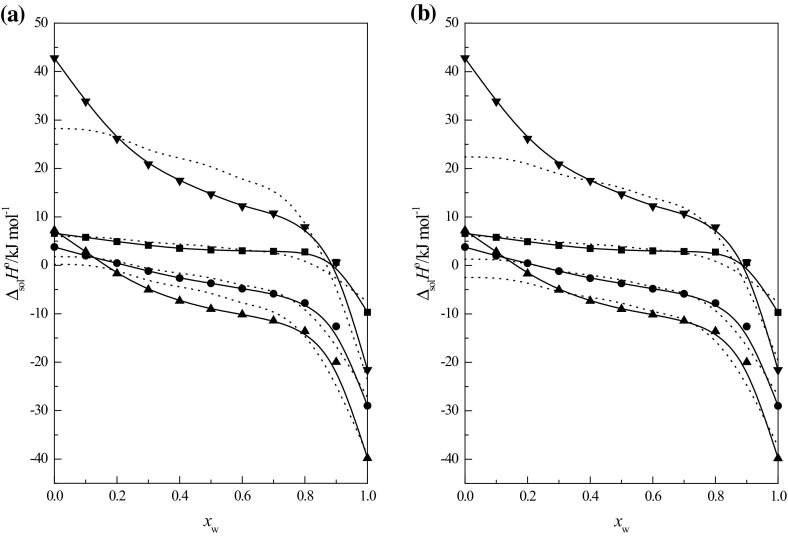
The standard enthalpy of solution of 1,4-dioxane (*filled square*), 12C4 (*filled circle*), 15C5 (*filled triangle*) (data from Ref. [31] and 18C6 (*filled inverted triangle*) in ${\text{EtOH}} + {\text{W}}$ mixtures at *T* = 298.15 K as a function of *x*_w_, experimental data (solid line) and data calculated using the Eq. 8 (*dotted line*): **a** using the data of standard solution enthalpy of cyclic ethers for *x*_w_ = 0, 0.1, 0.2, 0.3, 0.4, 0.5, 0.6, 0.7, 0.8, 0.9 and 1; **b** using the data of standard enthalpy of solution of cyclic ethers for *x*_w_ = 0.3, 0.4, 0.5, 0.6, 0.7, 0.8, 0.9 and 1

It was also observed that the regression coefficient (*R* ^2^) of Eq. 8 is the highest for 12C4 but those for 1,4-dioxane, 15C5 and 18C6 *R* ^2^ are much lower. This means that in these cases Eq. 8 is not well satisfied. The standard enthalpy of solution of cyclic ethers was recalculated using Eq. 8 and the parameters are given in Table 4. The results obtained are shown in Fig. 3a. As is seen, the courses of function $\Delta_{\text{sol}} H^{\text{o}}$ = *f*(*x*_w_), calculated with the use of Eq. 8 and that obtained experimentally, clearly differ in the case of 15C5 and 18C6. This is probably due to the preferential solvation of 15C5 and 18C6 molecules by water molecules.

It was decided to make a recalculation of the enthalpy of solution of cyclic ethers using Eq. 8 while omitting the data related to the mixtures with a high alcohol content, *i.e. x*_w_ = 0, *x*_w_ = 0.1 and *x*_w_ = 0.2. The results obtained are given in Table 4 (columns labeled b) and recalculated functions $\Delta_{\text{sol}} H^{\text{o}} = f (x_{\text{w}} )$ for cyclic ethers are show in Fig. 3b. As is seen in Table 4, the regression coefficient *R* ^2^ is significantly higher for 15C5 and 18C6. As shown in Fig. 3b, the shapes of function $\Delta_{\text{sol}} H^{\text{o}} = f (x_{\text{w}} )$ calculated using the parameters of Eq. 8 are very similar to the curves obtained experimentally in the range of high and medium water contents. This means that the acidic properties of the ${\text{EtOH}} + {\text{W}}$ mixtures significantly affect the dissolution enthalpy of cyclic ethers in these mixtures. One can still observe a substantial deviation of the calculated function from that obtained by experiment for 15C5 and 18C6 in the range of high ethanol content in the mixtures. In my opinion this is probably due to the preferential solvation of their molecules by water molecules (see above).

Moreover, as stated previously the values of coefficient *a* increase linearly with the increase of size of the cyclic ring (Eq. 10), *i.e.* with the increase of number of oxygen atoms ($n_{\text{ - O - }}$) in the molecules, but with a much higher regression coefficient (*R* ^2^) and much lower standard deviation (*SD*):

10 $$ a = { 1}. 1 3\left( { 1. 2 6} \right){-} 20. 8 5\left( {0. 2 8} \right) \times n_{{ - {\text{O}} - }} ,\;{{R}}^{ 2} = \, 0. 9 9 9 6 4,\;SD = \, 0. 8 2 5 1,P = { 6}. 6 5\times 10^{ - 3} $$

**Fig. 4:**
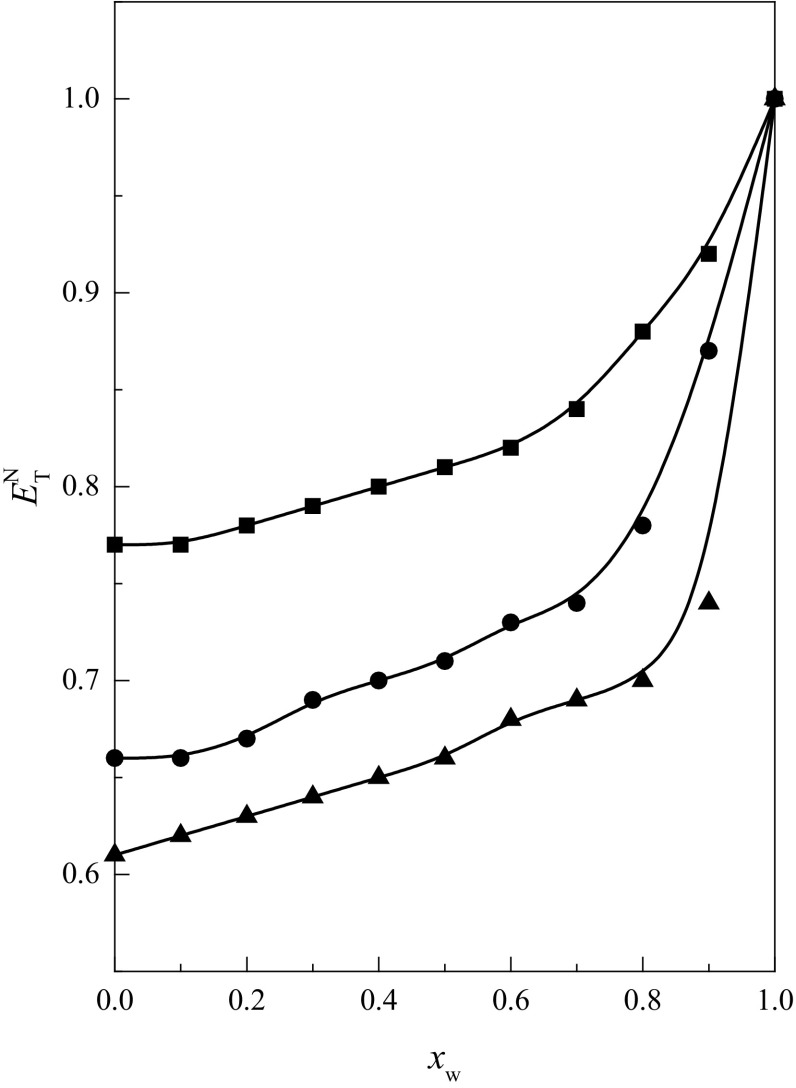
Acid properties expressed by the standardized Dimroth–Reichardt’s parameter $E_{\text{T}}^{\text{N}}$ for: ${\text{MeOH}} + {\text{W}}$ (*filled square*), ${\text{EtOH}} + {\text{W}}$ (*filled circle*) and ${\text{PrOH}} + {\text{W}}$ (*filled triangle*) (data from Ref. [26]) as a function of *x*_w_ at *T* = 298.15 K

In Table 5, the parameters of Eq. 8 for cyclic ethers in the ${\text{MeOH}} + {\text{W}}$, ${\text{EtOH}} + {\text{W}}$ and ${\text{PrOH}} + {\text{W}}$ mixtures are shown. As is seen, the values of the *Q*_0_ parameter are positive and decrease with increasing alcohol chain length, but the values of the *a* parameter are negative and increase (become less negative) with increasing alcohol chain length. Linear dependences $Q_{\text{o}} = f (n_{{ - {\text{C}} - }} )$ and $a = f (n_{{ - {\text{C}} - }} )$ (where *n*_–C–_ is the number of carbon atom is the alcohol molecule) were not observed. The highest values of $Q_{0}$ and *a* parameters were obtained for cyclic ethers in the ${\text{MeOH}} + {\text{W}}$ mixtures. This is consistent with the coefficient of acidity ($E_{\text{T}}^{\text{N}}$) values, which are the highest in the ${\text{MeOH}} + {\text{W}}$ mixtures (Fig. 4).

**Table 5:** Values of parameters of Eq. 8 for cyclic ethers in the ${\text{MeOH}} + {\text{W}}$, ${\text{EtOH}} + {\text{W}}$ and ${\text{PrOH}} + {\text{W}}$ mixtures at *T* = 298.15 K

	1,4-Dioxane	12C4	15C5	18C6
	*Q*_0_ (kJ·mol^−1^)			
MeOH + W^a^	48.54 (3.26)^d^	89.22 (3.36)^e^	109.00 (5.67)^e^	156.80 (5.96)^e^
EtOH + W^b^	32.22 (3.259)^d^	58.37 (3.64)^d^	65.30 (5.92)^e^	104.03 (6.53)^e^
PrOH + W^c^	26.90 (0.46)^e^	40.46 (3.58)^e^	46.34 (4.17)^e^	77.81 (5.34)^e^
	*a* (kJ·mol^−1^)			
MeOH + W^a^	–56.83 (3.90)^d^	–117.51 (3.91)^e^	–147.54 (6.59)^e^	–178.57 (6.93)^e^
EtOH + W^b^	–39.79 (4.31)^d^	–85.65 (4.84)^d^	–102.68 (7.55)^e^	–123.68 (8.33)^e^
PrOH + W^c^	–36.65 (0.64)^e^	–70.33 (4.91)^e^	–87.99 (5.72)^e^	–100.13 (7.35)^e^
	*R* ^2^			
MeOH + W^a^	0.95936^d^	0.99341^e^	0.98818^e^	0.99104^e^
EtOH + W^b^	0.90432^d^	0.97211^d^	0.96855^e^	0.97352^e^
PrOH + W^c^	0.99820^e^	0.97157^e^	0.97526^e^	0.97373^e^

^a^Data taken from Ref. [24]

^b^Data taken from the present paper

^c^Data taken from Ref. [25]

^d^The parameters of Eq. 8 calculated using the standard solution enthalpy data of cyclic ethers for *x*_w_ = 0, 0.1, 0.2, 0.3, 0.4, 0.5, 0.6, 0.7, 0.8, 0.9 and 1

^e^The parameters of Eq. 8 calculated using the of standard solution enthalpy data of cyclic ethers for *x*_w_ = 0.3, 0.4, 0.5, 0.6, 0.7, 0.8, 0.9 and 1

## Conclusion

The contribution of the –CH_2_CH_2_O– group to the standard solvation enthalpy of cyclic ethers in water is much higher than that in ethanol. This is connected with hydrophobic hydration of cyclic ethers in water.

Based on the analysis of the preferential solvation model proposed by Waghorne, it can be concluded that the cyclic ethers are preferentially solvated by water molecules over the whole range of the solvent mixture compositions. 12C4, 15C5 and 18C6 molecules are hydrophobicaly hydrated. The interactions of 1,4-dioxane with water molecules are different. Probably, hydrogen bonds are formed between water and 1,4-dioxane molecules.

In the ${\text{EtOH}} + {\text{W}}$ mixtures the solution enthalpy of 12C4 depends on the acidic properties of this mixed solvent over the whole range of the mixture composition. In the case of 1,4-dioxane, 15C5 and 18C6 this dependency is observed for solutions in the range of medium and high water content. 
